# Dietary Inflammatory Index and Cardiovascular Disease Risk in Australian Adults: A Secondary Analysis of the OLIVAUS Trial

**DOI:** 10.3390/nu18111732

**Published:** 2026-05-28

**Authors:** Jocelynne Young, Elena S. George, Wolfgang Marx, Hannah L. Mayr, James R. Hebert, Sherry Price, Colleen J. Thomas, Catherine Itsiopoulos, George Moschonis, Yingting Cao, Katerina Sarapis

**Affiliations:** 1 Department of Food, Nutrition and Dietetics, School of Allied Health, Human Services and Sport, La Trobe University, Melbourne 3086, Australia; jojoycq@outlook.com (J.Y.); g.moschonis@latrobe.edu.au (G.M.); 2 Institute for Physical Activity and Nutrition (IPAN), School of Exercise and Nutrition Sciences, Deakin University, Geelong 3220, Australia; elena.george@deakin.edu.au; 3 IMPACT (The Institute for Mental and Physical Health and Clinical Translation), Food & Mood Centre, Deakin University, Geelong 3220, Australia; wolf.marx@deakin.edu.au; 4 Department of Nutrition and Dietetics, Princess Alexandra Hospital, Woolloongabba 4102, Australia; hannah.mayr@health.qld.gov.au; 5 PA-Southside Clinical Unit, Faculty of Health, Medicine and Behavioural Sciences, The University of Queensland, St Lucia 4072, Australia; 6 Department of Epidemiology and Biostatistics and Cancer Prevention and Control Program, Arnold School of Public Health, University of South Carolina, Columbia, SC 29206, USA; jhebert@mailbox.sc.edu (J.R.H.); sherryp@email.sc.edu (S.P.); 7 Department of Nutrition, Connecting Health Innovations LLC (CHI), Columbia, SC 29201, USA; 8 Department of Microbiology, Anatomy, Physiology and Pharmacology, School of Agriculture, Biomedicine and Environment, La Trobe University, Bundoora 3086, Australia; colleen.thomas@latrobe.edu.au; 9 College of Science Technology Engineering Mathematics (STEM), RMIT University, Melbourne 3000, Australia; catherine.itsiopoulos@rmit.edu.au; 10 Department of Nutrition & Dietetics, Harokopio University, 17671 Athens, Greece; 11 Non-Communicable Diseases and Implementation Science, Baker Heart and Diabetes Institute, Melbourne 3004, Australia

**Keywords:** cardiovascular risk factors, energy-adjusted dietary inflammatory index, inflammation, diet, polyphenol, olive oil

## Abstract

**Background:** The Dietary Inflammatory Index (DII^®^) is a commonly used tool to assess diet-related inflammation. Higher DII scores have been associated with increased cardiovascular disease risk in observational studies. However, evidence examining cardiovascular outcomes across DII levels in controlled settings remains limited. This secondary analysis examined cross-sectional differences and longitudinal associations between dietary inflammatory potential and cardiovascular outcomes in healthy Australian adults. **Methods:** This study used data from a double-blind randomised crossover trial, in which 50 participants consumed 60 mL/day of either extra virgin high-polyphenol olive oil (HPOO; 320 mg/kg) or low-polyphenol olive oil (LPOO; 86 mg/kg) across two 3-week intervention periods, separated by a 2-week washout. Anthropometric measures (weight, height, waist circumference, and BMI) and cardiovascular outcomes (i.e., blood pressure, lipids, oxidised LDL, and HDL cholesterol efflux capacity) were assessed at four timepoints. DII and energy-adjusted DII (E-DII^TM^) scores were derived from 3-day food diaries. Linear mixed-effects models were used to compare cardiovascular outcomes across repeated-measures DII tertiles (low, medium, and high), adjusting for intervention, period, sequence, age, sex and waist circumference. **Results:** Forty-three participants completed this study. At baseline, BMI, waist circumference, systolic blood pressure, total cholesterol, and LDL differed significantly across DII tertiles (*p* < 0.05). However, over time, cardiovascular outcomes did not differ between medium or high versus low DII tertiles, and no significant time-by-tertile interactions were observed (all *p* > 0.05). DII values remained stable, while E-DII showed modest within-person reductions during both intervention periods (mean reduction: 0.886 units vs. 0.596 units). **Conclusions**: In this healthy cohort, there was no evidence of a consistent association between DII and short-term differences in cardiovascular outcomes across the intervention period. These findings should be interpreted cautiously, given the observational nature of DII groupings. Longer-duration studies with greater variation in dietary inflammatory potential are warranted to clarify the relationship between DII and cardiovascular health.

## 1. Introduction

Cardiovascular disease (CVD) remains one of the leading causes of morbidity and mortality worldwide and in Australia [1,2]. In 2022, over 4.5 million Australians self-reported living with CVD, and it accounted for approximately 24% of all deaths, with more than 1500 hospitalisations each day [2,3]. This burden underscores the need to identify modifiable determinants of early CVD risk and to implement effective prevention strategies across the life course.

Chronic, low-grade systemic inflammation is widely recognised as a key pathophysiological contributor to the initiation and progression of CVD. Persistent inflammatory signalling can disrupt vascular homeostasis by promoting endothelial dysfunction and reducing nitric oxide bioavailability, while accelerating oxidative modification of lipoproteins and facilitating atherogenesis and plaque instability [4]. Over time, these processes can impair lipid metabolism, blood pressure regulation, and oxidative balance, collectively contributing to cardiovascular risk [5].

Multiple factors contribute to systemic inflammation, including genetic, environmental, and lifestyle factors. Among these, long-term adherence to specific dietary patterns has been shown to either exacerbate or attenuate systemic inflammation, thereby influencing cardiovascular risk [6,7]. Diets high in saturated fats, refined grains, added sugars, and ultra-processed foods have been associated with metabolic dysfunction and higher concentrations of pro-inflammatory biomarkers, including C-reactive protein (CRP), and related cytokine markers [8,9,10]. In contrast, diets rich in vegetables, fruits, whole grains, legumes, nuts, and unsaturated fats are generally associated with lower inflammation and improved vascular health [9,11]. The Mediterranean dietary pattern is one of the most extensively studied examples and includes functional foods rich in bioactive compounds, such as olive oil, nuts, fruits, vegetables and legumes. Extra virgin olive oil (EVOO) is a key source of fat in the Mediterranean diet (MedDiet) and is rich in phenolic compounds, including hydroxytyrosol, tyrosol and oleuropein derivatives. These bioactive compounds exhibit antioxidant and anti-inflammatory properties and are thought to support endothelial function and cardiovascular health. Evidence from intervention and observational studies suggests that anti-inflammatory dietary patterns, including the MedDiet, are associated with favourable cardiovascular markers, including blood pressure, lipid profile, and hs-CRP [11,12]. Overall, this growing body of evidence suggests that diet quality plays an important role in modulating inflammation and may influence CVD risk [13].

The Dietary Inflammatory Index (DII^®^) was developed to quantify the inflammatory potential of an individual’s diet. It is a literature-derived score that incorporates up to 45 food and nutrient parameters identified as having pro- or anti- inflammatory associations with circulating inflammatory biomarkers. The DII was initially validated against hs-CRP, demonstrating its ability to predict elevated (>3 mg/L) concentrations [9]. It has since been associated with other inflammatory markers in larger observational studies. Higher DII scores reflect more pro-inflammatory dietary patterns, typically characterised by higher saturated fat and refined carbohydrate intake and lower consumption of fruits, vegetables, and nutrient-dense foods [9]. In observational studies, dietary patterns with more pro-inflammatory DII profiles have been associated with adverse cardiovascular outcomes, including higher CVD incidence and mortality. For example, in the SUN cohort—a longitudinal study of Spanish university graduates—individuals in the highest DII quartile had approximately twice the risk of developing CVD compared with the lowest quartile, and higher DII categories have also been associated with increased all-cause mortality [13,14]. Conversely, lower (more anti-inflammatory) DII scores are commonly observed in those following healthier dietary patterns such as low-fat diets and Mediterranean-style eating patterns [15,16].

Despite these associations, important gaps remain in the literature regarding DII and CVD outcomes. Most evidence derives from observational studies, limiting the ability to assess within-individual changes in dietary inflammatory potential alongside corresponding changes in cardiovascular markers over time [17,18]. Whole-diet intervention studies specifically designed to modify the DII are still limited. In this context, analyses conducted within controlled trial settings may provide meaningful insights. Repeated measurements and rigorous outcome assessment may strengthen temporal relationships and reduce measurement error, although such analyses remain observational when dietary inflammatory potential is not directly manipulated.

Using data from a randomised, crossover trial (the OLIVAUS study), originally designed to investigate the effects of olive oil polyphenols on CVD markers, this secondary analysis aims to examine longitudinal associations between dietary inflammatory potential that are derived from the whole diet using a 3-day food diary and cardiovascular outcomes (including BP, hsCRP, oxidative stress, and lipid metabolism) in healthy adults. DII and E-DII tertiles were derived post hoc from dietary records and were not randomised exposures; therefore, analyses of the DII should be interpreted as observational within the context of a controlled trial.

## 2. Materials and Methods

The present study is a secondary analysis of data collected in a 10-week double-blind, randomised, controlled crossover trial (OLIVAUS) designed to explore the effects of extra virgin high-polyphenol olive oil (HPOO) versus low-polyphenol olive oil (LPOO) on cardiovascular risk markers in healthy Australian adults [19,20]. The original study was conducted in accordance with Good Clinical Practice guidelines, the Declaration of Helsinki, and reported in accordance with CONSORT guidelines standards. All procedures were approved by the La Trobe University Human Research Ethics Committee (HEC17-067). The trial was prospectively registered with the Australia New Zealand Clinical Trials Registry (ACTRN12618000706279, 30 April 2018), and written informed consent was obtained from all participants.

### 2.1. Study Participants

Volunteers were recruited in Melbourne, Australia, via social media, La Trobe University email databases, word of mouth, and campus posters. Eligibility was determined using a standardised screening procedure. Participants were required to be aged 18–75 years with a body mass index (BMI) between 18.5 and 40 kg/m^2^. Exclusion criteria included non-English-speaking individuals, pregnant or lactating women, smokers, those following medically prescribed special diets (e.g., gluten-free for coeliac disease), and individuals with high habitual olive oil intake (>1 tablespoon/day). Participants were also excluded if they regularly consumed vitamin or antioxidant supplements and were unable to cease them for the study duration (except iron, calcium, and vitamin D), or if they were taking prescribed medications such as antihypertensive agents, lipid-lowering drugs, or non-steroidal anti-inflammatory drugs. Additional exclusions included diagnosed chronic disease (e.g., diabetes, hyperlipidaemia, hypertension, and inflammatory conditions), gastrointestinal disease, or any other condition likely to impair adherence to the protocol.

### 2.2. Study Design and Procedure

Participants were randomly assigned to one of two intervention sequences (HPOO vs. LPOO). The intervention comprised two 3-week periods (Period 1 and Period 2). During the first intervention period (Period 1; timepoints T1 to T3), participants were asked to consume 60 mL/day of either HPOO (320 mg/kg phenolics) or LPOO (86 mg/kg phenolics), incorporated raw into their usual diet. Following a 2-week washout period, participants crossed over to consume the alternate oil for a further 3 weeks (Period 2; timepoints T4 and T6). The test oils were stored in identical sealed containers with concealed codes to maintain blinding of participants and investigators. Codes were revealed only after completion of statistical analyses.

Full protocol details, including randomisation procedures, sample size, dietary and physical activity procedures, anthropometry, compliance, blinding, and adverse event reporting, are reported in the original OLIVAUS study protocol [19,20,21].

### 2.3. Sample Characteristics

At screening and baseline, socio-demographic information was collected, including age, sex, languages spoken at home, education level, ethnicity, and parental country of birth. Anthropometric and lifestyle data (e.g., weight, height, waist circumference, and smoking status), as well as medical history, medication use, and dietary supplement use, were also recorded. Waist circumference was included as an adiposity-related covariate in the adjusted models because central adiposity is closely associated with cardiometabolic risk markers and has been considered in previous DII and cardiometabolic risk research [22]; however, as it may lie on the causal pathway between dietary inflammatory potential and cardiometabolic outcomes, results should be interpreted with consideration of potential over-adjustment.

### 2.4. Measurements

#### 2.4.1. Dietary Intake Assessment

Dietary intake was assessed using a 3-day food diary collected at baseline (T1 and T4) and at the end of each intervention period (T3 and T6), as described in the trial protocol [20]. Participants recorded all foods and beverages consumed over two weekdays and one weekend day (preferably non-consecutive), including portion sizes, brands, preparation methods, and cooking techniques. Guidance on diary completion and incorporation of the trial oils in raw, uncooked form was provided at the pre-baseline visit. Diaries were reviewed at assessment visits for completeness and accuracy.

Nutrient analyses (energy and macro- and micronutrients) were performed using FoodWorks^®^ 9 (Xyris Software Pty Ltd., Brisbane, QLD, Australia) and relevant databases (Australia—AusFoods 2017, Aus Brands 2017, AUSNUT 2011–2013). To characterise intake of phenolic-rich foods during the intervention, data for relevant phenolic classes (including flavonoids, lignans, total polyphenols, other polyphenols, and stilbenes) were extracted from dietary records. The Phenol-Explorer database was used to estimate polyphenol composition of foods [23]. Average phenolic concentrations from the database were applied to calculate phenolic content of specified food groups for inclusion in analyses.

#### 2.4.2. Dietary Inflammatory Index (DII^®^) and Energy-Adjusted DII (E-DII^TM^)

In the present study, the inflammatory potential of the diet was calculated based on the Dietary Inflammatory Index (DII^®^), which was developed to quantify the inflammatory potential of individuals’ diets on a scale from maximally anti-inflammatory (most negative score) to maximally pro-inflammatory (most positive score). The development of the DII has been described by Shivappa et al. (2014) [24]. Briefly, the DII scoring algorithm was based on a careful review of the literature, through which 1943 articles identified 45 food parameters (i.e., macronutrients including specific categories of fatty acids, carbohydrate, and proteins; micronutrients including vitamins and minerals; flavonoids; and whole food items, including herbs and spices) as having sufficiently robust literature in relation to six inflammatory biomarkers, i.e., interleukins (IL)-1b, -4, -6, and -10, TNFα, and CRP [25,26].

In this study, self-report values for 29 of these food parameters were available from the 3-day food diaries. These were translated into z-scores using a global comparative database consisting of data from 11 countries by subtracting from the individual’s self-report value the mean of the global database and then dividing by the standard deviation. These scores were then converted to proportions (i.e., with values ranging from 0 to 1) and centred on zero by doubling each and subtracting 1 (2 × proportion − 1). These centred proportions were then multiplied by their respective coefficients (overall food parameter-specific inflammatory effect scores) to obtain DII scores for each food parameter. These were summed to obtain the overall DII score at T1, T3, T4, and T6.

Energy-adjusted DII (E-DII^TM^) scores were calculated using the density approach by calculating the DII per 1000 kcal consumption. This employed the same procedure for scoring but relied on an energy-adjusted global comparison database [27,28]. These DII and E-DII scores have a potential range from approximately −9 to +8, i.e., from minimally to maximally pro-inflammatory, respectively. The DII and E-DII are scored similarly and scaled identically; so, the scores are comparable across studies [27]. The DII/E-DII has been construct-validated in many (>60) studies, including one meta-analysis on CRP [29].

For this study, the following 29 of the 45 food parameters were used to calculate an individual’s overall DII score: energy, protein, total fat, saturated fat, trans fat, monounsaturated fatty acids (MUFAs), polyunsaturated fatty acids (PUFAs), cholesterol, carbohydrate, alcohol, fibre, thiamine, riboflavin, niacin, vitamin C, vitamin E, vitamin B6, vitamin B12, vitamin A, folic acid, beta-carotene, magnesium, iron, zinc, selenium, omega-6, omega-3, caffeine, and mercury. For the E-DII, energy was in the denominator; therefore, 28 parameters were used for computation. The decision to use the DII or E-DII was based on model goodness of fit or overall model explanatory ability.

In the present analysis, the DII was selected as the primary exposure to reflect absolute dietary inflammatory potential. However, given that total energy intake may vary within individuals over time, the E-DII was also examined in secondary analyses. Both metrics are presented to provide a comprehensive assessment of dietary inflammatory potential.

#### 2.4.3. Biochemical Analyses

Fasting venous blood samples were collected in the early morning at baseline (T1, T4) and at the end of each intervention period (T3, T6) following a 10 h fast. Blood was centrifuged (Hettich Rotina 420R, Beverly, MA, USA) at 2350 rpm for 10 min at 4 °C. Serum and/or plasma were aliquoted into 500 μL volumes and stored at −80 °C until analysis [21].

Total cholesterol and triglycerides were measured using the Alinity c Cholesterol Assay and Alinity c Triglyceride Assay, respectively (Abbott GmbH & Co., Wiesbaden, Germany). LDL cholesterol was measured using a direct quantitative method (Alinity c Direct LDL Assay; Sekisui Diagnostics, Charlottetown, Canada) and HDL cholesterol using a homogeneous method (Ultra HDL Assay; Abbott GmbH & Co., Wiesbaden, Germany). Oxidised LDL was measured using a solid-phase, two-site enzyme-linked immunosorbent assay (ELISA; Mercodia, Uppsala, Sweden). HDL-C efflux was measured using the Cholesterol Efflux Fluorometric Assay Kit (BioVision, Fremont, CA, USA). Finally, the Alinity c CRP Vario assay (SENTINEL CH, Milano, Italy) was used for the quantitative immunoturbidimetric determination of hs-CRP in human serum.

The methodology and primary results for these biochemical markers have been reported previously [19,30]. In the present secondary analysis, these biomarker data were re-analysed after stratifying participants according to tertiles of the DII score to examine whether cardiometabolic outcomes differed across levels of dietary inflammatory potential.

#### 2.4.4. Blood Pressure Measurements

Blood pressure (BP) was measured using applanation tonometry with a SphygmoCor XCEL system (AtCor Medical, Sydney, NSW, Australia) at baseline (T1 and T4) and at the end of each intervention period (T3 and T6) [20]. After a minimum 5 min rest in the supine position, peripheral/brachial systolic and diastolic BP were measured using an appropriately sized cuff placed on the upper left arm. Three consecutive recordings were obtained, and the average of the final two measurements was used for analysis. Central (aortic) SBP, DBP, and pulse pressure (PP) were derived from the brachial BP cuff [20]. Participants were classified using the American College of Cardiology (ACC) and American Heart Association (AHA) BP categories (normal, elevated, stage I hypertension, and stage II hypertension).

#### 2.4.5. Statistical Analysis

The DII and E-DII were analysed as continuous variables across four timepoints (T1, T3, T4, and T6) and were also categorised into tertiles. For baseline descriptive analyses, participants were categorised according to their T1 DII score. For longitudinal mixed-effects models, tertiles were defined using all repeated DII observations across T1, T3, T4 and T6. Thus, tertiles reflect a time-varying exposure, and individual participants could contribute observations to different DII tertiles across timepoints. Descriptive statistics are presented as means ± standard deviation (SD) for continuous variables and as counts and percentages for categorical variables. Baseline differences across T1 DII tertiles were assessed using one-way ANOVA for continuous variables and χ^2^ tests or Fisher’s exact tests for categorical variables, as appropriate. The *p*-values in Table 1 represent omnibus comparisons across the three baseline DII tertiles. Within-person changes during each intervention period were initially explored using paired t-tests comparing pre- and post-period values (Period 1: T1 to T3; Period 2: T4 to T6). Period-specific change scores were calculated as Δ = end − baseline for each period. Between-sequence differences in period-specific and overall change scores were assessed using independent *t*-tests. These *t*-tests and change-from-baseline analyses were considered exploratory. Assumptions of normality and homogeneity of variance were assessed, and non-parametric tests were used in sensitivity analyses where appropriate. Analyses were repeated for the E-DII.

The primary analyses used linear mixed-effects models fitted to long-format data to examine longitudinal associations while accounting for the crossover design and repeated measurements. Mixed-effects models were estimated using restricted maximum likelihood (REML), which allows inclusion of incomplete repeated-measures data under the missing-at-random assumption. Separate models were constructed for each cardiometabolic outcome (average systolic and diastolic BP, total cholesterol, triglycerides, HDL cholesterol, LDL cholesterol, oxidised LDL, and HDL cholesterol efflux capacity). For the mixed-effects models, repeated DII observations were categorised into low, medium and high tertiles using data-driven cut-off points that divided the 183 observations into approximately equal-sized groups (*n* = 61 per tertile) irrespective of baseline participant grouping. In the mixed-effects models, DII tertile, timepoint, period, intervention and sequence were specified as fixed effects, with participant included as a random effect to account for within-person correlation due to repeated measures. Timepoint was treated as categorical. Models estimated adjusted differences in outcomes between DII tertiles averaged across the study period, with β coefficients and 95% confidence intervals reported. Timepoint × tertile interactions were examined to assess whether associations differed across measurement occasions. Models were estimated using restricted maximum likelihood (REML), allowing participants with incomplete repeated-measures data to contribute available observations under the assumption that data were missing at random. Unadjusted models were fitted initially, followed by adjustment for age, sex, and waist circumference. As hsCRP is a key inflammatory biomarker relevant to DII construct validity, linear mixed-effects models were used to examine the association between DII tertiles and hsCRP across repeated measures, using the same model structure as the primary cardiovascular outcomes. Sensitivity analyses were also conducted, excluding waist circumference from the adjusted models to assess the potential influence of adiposity adjustment on the observed associations. Statistical significance was set at *p* < 0.05. All analyses were conducted using Jamovi version 2.7.9 [31].

## 3. Results

**Figure 1:**
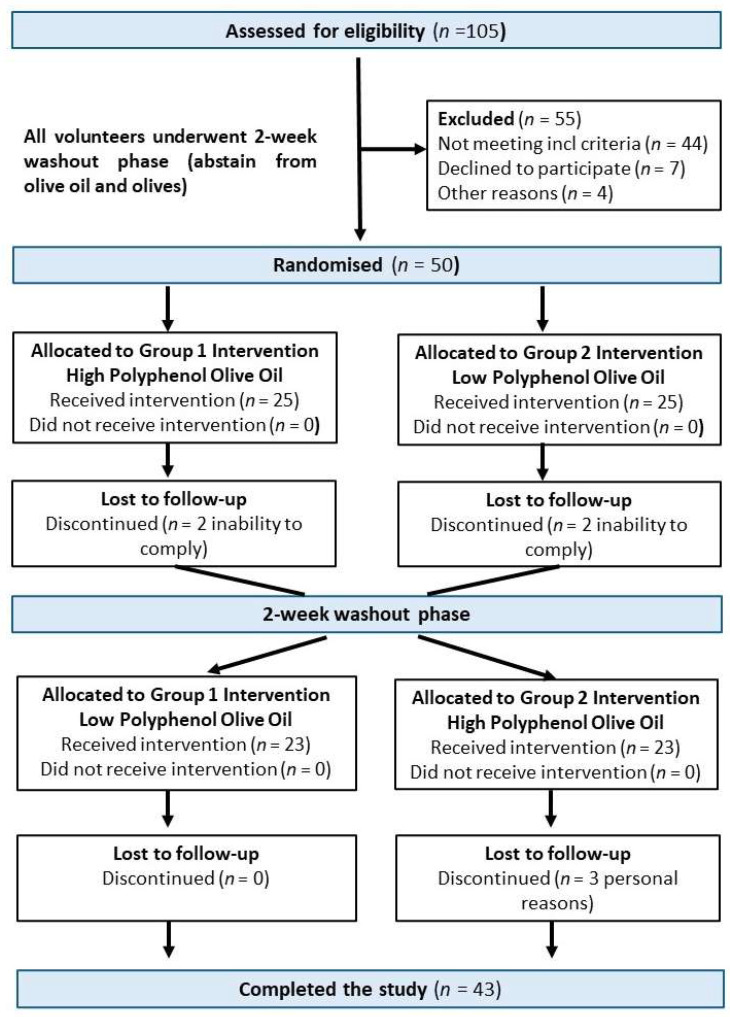
OLIVAUS study participant flow diagram.

Of the 105 volunteers who expressed interest, 50 eligible individuals (aged 38.5 ± 13.9 years; 66% females) were recruited and randomly allocated to either the LPOO (*n* = 25) or HPOO (*n* = 25) sequence. At the end of the 10-week intervention, 43 participants completed this study (LPOO sequence, *n* = 23; HPOO sequence, *n* = 20). The participant flow diagram (Figure 1) and the primary trial outcomes have been reported previously [20].

Baseline characteristics by DII tertile are presented in Table 1. Across tertiles, age, height, and weight did not differ significantly (all *p* > 0.05). Significant differences across DII tertiles were observed for BMI (*p* = 0.008), waist circumference (*p* = 0.003), peripheral SBP (*p* = 0.043), total cholesterol (*p* = 0.043), and LDL cholesterol (*p* = 0.035). Central DBP, peripheral DBP, HDL cholesterol, oxidised LDL, and HDL cholesterol efflux capacity were similar across tertiles, while triglycerides and central SBP showed non-significant trends across tertiles (*p* = 0.060 and *p* = 0.080, respectively). Total cholesterol and LDL cholesterol differed significantly across DII tertiles (total cholesterol *p* = 0.043; LDL cholesterol *p* = 0.035) (Table 1). Central DBP, peripheral DBP, HDL cholesterol, oxidised LDL, and HDL cholesterol efflux capacity were similar across tertiles, while triglycerides and central SBP showed non-significant trends across tertiles (*p* = 0.060 and *p* = 0.080, respectively).

**Table 1:** Baseline (T1) characteristics and cardiovascular parameters of OLIVAUS participants by DII tertiles.

	Tertile 1: Low DII DII ≤ −1.68 (*n* = 17)	Tertile 2: Medium DII −1.68 < DII ≤ −0.315 (*n* = 16)	Tertile 3: High DII DII > −0.315 (*n* = 17)	*p*-Value
Age (years)	33.1 (12.2)	41.4 (14.2)	41.2 (14.4)	0.127
Height (m)	1.72 (0.0968)	1.70 (0.108)	1.65 (0.0715)	0.057
Weight (kg)	67.9 (11.1)	76.5 (13.9)	67.9 (12.1)	0.125
BMI (kg/m^2^)	22.9 (2.67)	26.4 (3.31)	24.9 (3.56)	0.008 *
Waist circumference (cm)	80.6 (6.85)	91.2 (9.60)	88.6 (13.9)	0.003 *
**Gender (%)**				
Females	58.8%	56.3%	82.4%	
Males	41.2%	43.8%	17.6%	
Education (years)	16.1 (1.96)	17.6 (3.18)	18.1 (4.71)	0.144
**Educational level (%)**				
Secondary	0.0%	6.3%	5.9%	
Tertiary	88.2%	81.3%	88.2%	
Trade	5.9%	6.3%	0.0%	
Other	5.9%	6.3%	5.9%	
**English first language (%)**				
Yes	76.5%	75.0%	76.5%	
No	23.5%	25.0%	23.5%	
**Country of Birth (%)**				
Australia, NZ, Pacific Islanders	82.4%	75.0%	52.9%	
Europe	0.0%	0.0%	29.4%	
South America	0.0%	25.0%	0.0%	
Middle East and Asia	17.6%	0.0%	17.6%	
**Ethnicity (%)**				
Asian	23.5%	6.3%	23.5%	
Caucasian Australian	70.6%	68.8%	41.2%	
Caucasian European	0.0%	6.3%	35.3%	
Islander	5.9%	0.0%	0.0%	
Latin American	0.0%	18.8%	0.0%	
**Haemodynamic Indices**				
Peripheral SBP (mmHg)	115.0 (11.1)	127.2 (14.7)	118.3 (11.9)	0.043 *
Peripheral DBP (mmHg)	68.5 (7.17)	72.3 (9.79)	70.2 (8.04)	0.457
Central SBP (mmHg)	102.0 (9.92)	112.8 (15.6)	106.4 (12.6)	0.080
Central DBP (mmHg)	67.8 (7.58)	73.6 (10.2)	70.8 (7.85)	0.189
**Pathology**				
Triglycerides (mmol/L)	0.81 (0.32)	1.30 (0.84)	1.01 (0.33)	0.060
Total cholesterol (mmol/L)	4.59 (1.12)	5.21 (0.96)	5.45 (0.74)	0.043 *
HDL cholesterol (mmol/L)	1.55 (0.31)	1.49 (0.31)	1.64 (0.38)	0.471
LDL cholesterol (mmol/L)	2.63 (0.86)	3.19 (0.87)	3.37 (0.71)	0.035 *
Oxidised LDL (mU/mL)	73.0 (25.4)	76.7 (19.4)	81.8 (23.1)	0.575
HDL cholesterol efflux capacity (%)	51.7 (5.0)	53.6 (4.5)	53.8 (4.8)	0.407
hsCRP (mg/L)	1.00 (1.85)	1.86 (2.77)	2.59 (3.56)	0.239

Abbreviations: BMI, body mass index; SBP, systolic blood pressure; DBP, diastolic blood pressure; HDL, high-density lipoprotein; LDL, low-density lipoprotein; DII, dietary inflammatory index; hsCRP, high-sensitivity C-reactive protein; values are presented as means ± SD for continuous variables and *n* (%) for categorical variables; *p*-values represent omnibus comparisons across baseline T1 DII tertiles, assessed using one-way ANOVA for continuous variables and χ^2^ or Fisher’s exact tests for categorical variables, as appropriate; negative numbers reflect anti-inflammatory scores, while positive numbers reflect pro-inflammatory scores; * *p* < 0.05.

### Association Between DII Tertiles and Cardiovascular Profiles

The mixed-effects analyses included 183 repeated-measures observations across four timepoints (T1, T3, T4, and T6). Participant contributions at each timepoint were T1, *n* = 50; T3, *n* = 47; T4, *n* = 43; and T6, *n* = 43, reflecting discontinuation by seven participants. Reasons for withdrawal have been reported in the original OLIVAUS publication [30].

Repeated-measures DII values were grouped into low, medium and high tertiles to reflect the relative dietary inflammatory potential of each observation across all timepoints. Lower DII values indicate more anti-inflammatory dietary profiles, whereas higher DII values indicate more pro-inflammatory dietary profiles. Tertiles were defined by dividing the 183 repeated observations into three equal-sized groups (*n* = 61 per tertile). The DII cut-offs were tertile 1, DII ≤ −1.62; tertile 2, −1.62 < DII ≤ −0.180; and tertile 3, DII > −0.180. These repeated-measures tertiles were used for the longitudinal mixed-effects models and differ from the baseline T1 tertiles used in Table 1. Across the four timepoints of the study period, there were no significant differences in mean cardiovascular outcomes between medium versus low DII or high versus low DII (all *p* > 0.05) (Table 2). Relative to the low DII tertile, neither the medium nor high DII tertile was associated with statistically significant differences in peripheral or central BP, triglycerides, total cholesterol, HDL-cholesterol, LDL-cholesterol, oxidised LDL, or HDL-cholesterol efflux capacity (Table 2). In linear mixed-effects models, DII tertiles were not significantly associated with CRP across repeated measures. Given the inflammatory basis of the DII, associations between DII tertiles and hs-CRP were examined as a construct validity analysis. Overall, DII tertiles were not consistently associated with hs-CRP across repeated measures. Although one isolated contrast between the high and low DII tertiles reached statistical significance at T1 (*p* = 0.043), this association was not observed consistently across other time points and was, therefore, interpreted cautiously.

Overall, average cardiovascular levels were similar across DII tertiles (Table 3). Estimated peripheral SBP remained unchanged across tertiles (119 mmHg in the low and medium tertiles and 121 mmHg in the high tertile). Peripheral DBP was also comparable, with only a small difference in the high tertile (70.3 mmHg) compared with the low and medium tertiles (68.8 mmHg). Central SBP was unchanged across tertiles (106 mmHg in the low tertile and 107 mmHg in the medium and high tertiles). Central DBP was also stable, with a small increase in the high tertile (70.7 mmHg) compared with the low and medium tertiles (both 69.7 mmHg). Triglyceride levels also remained stable across all tertiles (low DII, 0.93 mmol/L; medium DII, 0.96 mmol/L; and high DII, 0.91 mmol/L). Total cholesterol and LDL cholesterol showed small variation between groups. HDL cholesterol was likewise similar (low DII, 1.55 mmol/L; medium DII, 1.60 mmol/L; high DII, 1.57 mmol/L). Oxidised LDL and HDL cholesterol efflux capacity did not differ meaningfully by DII tertile. Confidence intervals were overlapping, suggesting no clear changes in these outcomes across DII tertiles (Table 3). Additionally, time and DII tertile interactions showed no evidence of different cardiometabolic trajectories across low, medium, and high DII tertiles (all *p* > 0.05). Model-estimated marginal means of cardiovascular outcomes across tertiles of E-DII are presented in Supplementary Table S1.

Paired analyses were used to describe within-person changes in the DII across each intervention period. Unadjusted DII scores did not change within individuals in either period (Period 1, *p* = 0.484; Period 2, *p* = 0.576). In contrast, the E-DII decreased within individuals in both periods (mean reduction of 0.886 units in Period 1; *p* < 0.001) and 0.596 units in Period 2; *p* = 0.021), suggesting a modest shift in energy-adjusted dietary inflammatory potential (Supplementary Table S2). As the E-DII is standardised for energy intake, this change may reflect alterations in total energy intake and/or broader dietary behaviours during the intervention rather than substantial changes in the inflammatory composition of the diet. Corresponding changes in energy intake should be considered when interpreting these findings.

There was no evidence that treatment order influenced DII. Participants allocated to receive HPOO first versus LPOO first did not differ in DII scores at baseline or at any subsequent time point (all *p* > 0.05). In addition, the magnitude of the within-person DII change during Period 1 and Period 2 did not differ between sequences (*p* = 0.646 and *p* = 0.730, respectively). The average DII score change across both periods was also similar between intervention sequences (receiving HPOO vs. LPOO or receiving LPOO vs. HPOO) (*p* = 0.438) (Supplementary Table S3). Sensitivity analyses excluding waist circumference from the adjusted models produced broadly similar findings for DII, while some E-DII associations were attenuated (Supplementary Table S4).

## 4. Discussion

This secondary analysis investigated the relationship between the Dietary Inflammatory Index (DII) and CVD markers in healthy Australian adults participating in the OLIVAUS study [20]. We examined cross-sectional differences and longitudinal changes in CVD markers across DII tertiles. Our primary hypothesis, that higher DII scores would be associated with less favourable CVD markers and changes in CVD risk over time, was not supported by this secondary analysis. Because the E-DII is standardised per unit of energy intake, reductions in the E-DII may partly reflect changes in total energy consumption rather than true shifts in dietary inflammatory composition.

In this study, baseline anthropometric and cardiovascular measures varied across DII tertiles. Participants in the middle DII tertile had a notably higher BMI and waist circumference compared with those in the lowest DII tertile, suggesting that more pro-inflammatory dietary patterns may cluster with greater adiposity. This is relevant given that adiposity is a well-established contributor to chronic inflammation and metabolic risk. However, these differences did not show a clear stepwise pattern across DII tertiles, and higher DII scores do not necessarily imply that cardiometabolic risk markers would increase progressively across tertiles, as BMI, waist circumference and blood pressure are influenced by multiple biological, behavioural and lifestyle factors beyond dietary inflammatory potential. These baseline comparisons are descriptive and unadjusted, as the primary aim of this study was to assess longitudinal changes using mixed-effects models rather than test cross-sectional differences. However, the observed pattern is consistent with findings from the PREDIMED study, where higher (more pro-inflammatory) DII scores were independently associated with greater BMI and waist circumference after adjustment for confounders, including MedDiet adherence [22]. Although the analytical approaches differ, the consistency in direction strengthens the interpretation that pro-inflammatory dietary patterns tend to co-occur with higher adiposity.

Similarly, SBP also differed, peaking in the middle tertile in our baseline descriptive comparisons. A previous meta-analysis reported that being in the highest DII category was associated with a 1.2 mmHg significant increase in SBP and having higher odds of hypertension (OR: 1.13) [32]. Cross-sectional and case–control studies also support this relationship; one analysis found a 1.6-fold higher risk of hypertension in individuals with elevated DII, particularly in men [33]. These findings may provide context for the observed SBP differences, highlighting diet-induced inflammation as a potential contributor to elevated SBP.

Lipid profiles differed across tertiles, with total cholesterol and LDL cholesterol being higher in the middle and high DII groups compared with the low group. In this context, a meta-analysis investigating the relationship between DII scores and serum lipid profiles in adult populations found that individuals with the highest DII scores had elevated total cholesterol (+5.16 mg/dL) and LDL cholesterol (+3.99 mg/dL) compared with those in the lowest DII category. This supports the role of dietary inflammation in promoting an atherogenic lipid profile, aligning with our results in a distinct cohort [34]. However, it is important to acknowledge that our observations are based on baseline cross-sectional, unadjusted comparisons and are, therefore, hypothesis-generating only.

Although we observed some baseline differences across tertiles, the longitudinal mixed-effects models did not show consistent differences in changes over time. Consequently, our findings do not provide evidence for a clear longitudinal association between DII score and the assessed CVD risk outcomes [35]. Further longitudinal and interventional studies are needed to clarify temporal relationships between DII, cardiovascular outcomes and underlying inflammatory pathways.

In this study, no significant differences in cardiovascular profiles were observed across DII tertiles over the study period (all *p* > 0.05). These findings are broadly consistent with the main OLIVAUS paper, which also reported no significant differences between extra virgin HPOO and LPOO treatments in the total sample [20]. However, whereas the main OLIVAUS analysis demonstrated favourable within-arm changes following extra virgin HPOO consumption, particularly among participants with higher cardiometabolic risk, the present secondary analysis indicates that stratification by DII tertiles did not identify differential cardiovascular responses over the study period. Neither medium nor high DII tertiles were associated with changes in BP, lipid parameters, oxidised LDL, or HDL cholesterol efflux. These findings suggest that, despite baseline differences, dietary inflammatory potential did not translate into measurable cardiovascular effects during the intervention. Although the DII is designed to reflect the inflammatory potential of the diet and has been validated against biomarkers such as hsCRP, we did not observe a significant association between DII tertiles and hsCRP in this cohort. This may reflect the relatively narrow range of DII scores, the generally healthy study population with low baseline inflammation and the short duration of the intervention, which may limit the ability to detect changes in inflammatory markers. Therefore, the null hs-CRP findings should not be interpreted as evidence against the validity of the DII, but rather as a reflection of the constrained inflammatory and dietary exposure range in this study. The study design may have also attenuated differences. All participants consumed olive oil throughout this study, with one group receiving EVOO high in polyphenols (320 mg/kg) and the other consuming olive oil with a lower polyphenol concentration (86 mg/kg). Both oils contained polyphenols, which are known for their anti-inflammatory and antioxidant properties [30]. These compounds can improve endothelial function, reduce oxidative stress, and modulate lipid metabolism [30,36], potentially minimising variation across DII tertiles. Previous research shows that polyphenol-rich EVOO can reduce inflammatory markers, even in less favourable dietary contexts [30,37].

These findings highlight the complexity of diet–inflammation interactions and suggest that specific bioactive compounds may have contributed to attenuating potential differences. While the DII captures the inflammatory potential of the diet as a whole, the inclusion of potent anti-inflammatory foods such as polyphenol-rich olive oil may diminish its predictive value in intervention settings. Future studies should examine whether similar attenuation occurs with other anti-inflammatory dietary components and assess the long-term impact of polyphenol-rich interventions on cardiometabolic health [24,38]. Additionally, to our knowledge, intervention evidence directly linking the DII to cardiovascular outcomes remains limited, with most intervention studies focusing on short-term changes in inflammatory biomarkers rather than downstream cardiometabolic endpoints. For instance, the AUSMED Heart study examined diet-induced changes in DII scores in Australians with coronary heart disease and assessed inflammatory markers, including hs-CRP and IL-6, but no clinical endpoints [18]. This study reported associations between improvements in DII scores and IL-6 concentrations, but limited effects on other cardiometabolic biomarkers, including hs-CRP, adiponectin, and body composition. While IL-6 responds relatively rapidly to metabolic and oxidative stimuli, hs-CRP may be less sensitive to modest, short-term dietary changes [18]. An Umbrella review of systematic reviews and meta-analyses of dietary patterns and inflammation also showed strong effects on inflammatory biomarkers (i.e., CRP and IL-6), but noted insufficient evidence for downstream cardiometabolic outcomes due to the paucity of intervention studies [12].

In contrast, evidence linking DII to CVD outcomes largely comes from prospective observational studies with longer follow-up, where cumulative dietary inflammatory exposure can be captured. Several cohort studies have reported associations between higher DII scores and increased risk of CVD or mortality over multi-year follow-up periods [14,39,40]. These findings support the notion that dietary inflammatory potential may influence cardiovascular risk through longer-term, cumulative processes rather than short-term changes. In the OLIVAUS trial, each intervention period lasted only three weeks, which may not have been sufficient for differences in dietary inflammatory potential to translate into detectable changes in downstream cardiovascular outcomes such as lipid metabolism, vascular function, or HDL functionality. These outcomes are regulated by multiple physiological systems, including vascular tone and stiffness, renal sodium handling, autonomic regulation, and hepatic lipid metabolism, and are, therefore, less likely to respond to short-term dietary modulation [41,42,43].

These findings should be interpreted in light of this study’s strengths and limitations. A key strength is a rigorously conducted randomised, double-blind crossover design with repeated assessments. This design reduces confounding by controlling individual-level variability and improves efficiency in small samples [20]. Dietary intake was assessed at four timepoints (pre- and post-intervention) using detailed 3-day food diaries, which were reviewed for completeness, hence providing a stronger estimate of dietary exposure than single time-point assessments. The study also included a range of cardiometabolic outcomes, which assessed the overall impact on cardiovascular markers beyond inflammation alone.

However, several limitations should be acknowledged. The small sample size (*n* = 50) limits statistical power to detect subtle between-tertile differences after adjustments for key covariates. No separate power calculation was conducted for this secondary DII analysis, as the sample size was determined by the original OLIVAUS trial design. Given the potential for over-adjustment when including adiposity-related variables, additional sensitivity analyses excluding waist circumference from the adjusted models were conducted. Findings for the DII remained broadly consistent, whereas some E-DII associations were attenuated, suggesting that E-DII findings may be more sensitive to adiposity adjustment and model specification. These exploratory findings should, therefore, be interpreted cautiously. Larger sample sizes are generally required to detect associations [18,44]. The relatively short intervention periods may also have been insufficient for changes in dietary inflammatory potential to translate into detectable changes in cardiovascular markers. Although period and sequence were accounted for in the mixed-effects models, residual carryover effects inherent to the crossover design cannot be fully excluded. Although mixed-effects models using REML can accommodate incomplete repeated-measures data under the missing-at-random assumption, residual attrition bias cannot be excluded. Participant withdrawal during the second intervention phase may have contributed to some period or sequence imbalance in this relatively small crossover study. Furthermore, while 3-day food diaries are commonly used and repeated measures may strengthen exposure assessment, three days may be insufficient to capture full week-to-week variation, and self-reported intake is subject to reporting bias. In addition, the DII was calculated using 29 of the 45 possible food parameters; the remaining parameters could not be reliably derived from the available dietary data, and their influence on DII classification accuracy could not be formally assessed. This study was not originally designed to modify dietary inflammatory potential. Both groups received polyphenols from olive oil, although in different concentrations, which may have contributed to the relatively narrow range of DII scores in this cohort, with most participants exhibiting generally anti-inflammatory dietary patterns. This limited exposure contrast may have attenuated effect estimates and reduced the ability to detect associations. However, this interpretation remains hypothetical, as the present analysis was not designed to test whether olive oil polyphenols modified the association between the DII and cardiovascular outcomes. While analyses within a constrained exposure range can still provide useful insights, these findings should be interpreted cautiously and may not be generalisable to populations with more pro-inflammatory dietary patterns. Further studies specifically designed to modify DII across a wider range and over longer durations are warranted.

## 5. Conclusions

Overall, in this healthy, low-risk adult cohort, the DII was not associated with measurable short-term differences in cardiovascular outcomes across the intervention period. These findings suggest that within a relatively narrow and predominantly anti-inflammatory dietary range, variation in dietary inflammatory potential may not be sufficient to influence cardiovascular risk markers in the short term. However, given the observational nature of DII exposure, the restricted range of DII scores, and the short duration of follow-up, longer-term and specifically designed studies are required to better clarify the role of dietary inflammation in cardiovascular health.

## Figures and Tables

**Table 2:** Differences in cardiovascular outcomes comparing DII tertiles across the study period.

Cardiovascular Outcomes	Medium DII vs. Low DII β (95% CI)	*p*-Value	High DII vs. Low DII β (95% CI)	*p*-Value
Peripheral SBP (mmHg)	0.36 (−2.19, 2.91)	0.778	1.14 (−1.78, 4.06)	0.442
Peripheral DBP (mmHg)	0.00 (−1.97, 1.96)	0.996	1.49 (−0.74, 3.72)	0.190
Central SBP (mmHg)	0.60 (−1.76, 2.95)	0.619	1.19 (−1.43, 3.81)	0.372
Central DBP (mmHg)	0.07 (−1.86, 2.00)	0.943	1.04(−1.10, 3.185)	0.338
Triglycerides (mmol/L)	0.02 (−0.13, 0.18)	0.758	−0.02 (−0.20, 0.16)	0.826
Total cholesterol (mmol/L)	0.08 (−0.10, 0.26)	0.372	−0.08 (−0.29, 0.16)	0.440
HDL cholesterol (mmol/L)	0.05 (−0.01, 0.12)	0.101	0.03 (−0.05, 0.10)	0.460
LDL cholesterol (mmol/L)	0.05 (−0.09, 0.19)	0.518	−0.07 (−0.24, 0.09)	0.368
Oxidised LDL (mU/mL)	−4.03 (−12.01, 3.95)	0.320	3.58 (−4.96, 12.12)	0.408
HDL cholesterol efflux (%)	−0.20 (−1.15, 0.75)	0.679	−0.33 (−1.43, 0.77)	0.559
hsCRP (mg/L)	0.08 (−0.56, 0.72)	0.805	0.42 (−0.27, 1.11)	0.235

Abbreviations: SBP, systolic blood pressure; DBP, diastolic blood pressure; HDL, high-density lipoprotein; LDL, low-density lipoprotein; DII, Dietary Inflammatory Index; hsCRP, high-sensitivity C-reactive protein; β = adjusted mean difference for medium vs. low DII [31] and high vs. low DII. Models adjusted for age, gender, and waist circumference.

**Table 3:** Model-estimated marginal means of cardiovascular outcomes by DII tertile.

Cardiovascular Outcomes	Tertile 1 (Low DII)	Tertile 2 (Medium DII)	Tertile 3 (High DII)
Peripheral SBP (mmHg)	119 (116–123)	120 (117–123)	121 (117–124)
Peripheral DBP (mmHg)	68.8 (66.5–71.2)	68.8 (66.5–71.2)	70.3 (68.0–72.7)
Central SBP (mmHg)	106 (103–109)	107 (104–109)	107 (104–110)
Central DBP (mmHg)	69.7 (67.4–72.0)	69.7(67.5–72.0)	70.7 (68.4–73.0)
Triglycerides (mmol/L)	0.93 (0.753–1.11)	0.96 (0.781–1.13)	0.91 (0.731–1.09)
Total cholesterol (mmol/L)	4.97 (4.67–5.26)	5.05 (4.75–5.34)	4.88 (4.75–5.34)
HDL cholesterol (mmol/L)	1.55 (1.44–1.64)	1.60 (1.50–1.69)	1.57 (1.47–1.67)
LDL cholesterol (mmol/L)	3.01 (2.77–3.26)	3.06 (2.81–3.31)	2.94 (2.69–3.19)
Oxidised LDL (mU/mL)	72.1 (65.4–78.9)	68.1 (61.5–74.7)	75.7 (69.0–82.4)
HDL cholesterol efflux (%)	52.9 (51.5–54.4)	52.7 (51.3–54.2)	52.6 (51.1–54.1)
hsCRP (mg/L)	0.97 (0.38–1.56)	1.05 (0.48–1.62)	1.39 (0.80–1.98)

Abbreviations: SBP, systolic blood pressure; DBP, diastolic blood pressure; HDL, high-density lipoprotein; LDL, low-density lipoprotein; DII, Dietary Inflammatory Index; hsCRP, high-sensitivity C-reactive protein; values are estimated marginal means (95% confidence intervals) from linear mixed-effects models adjusted for intervention, period, sequence, age, sex, and waist circumference, averaged across the study period.

## Data Availability

The data presented in this study are available on request from the corresponding author due to privacy reasons.

## Supplementary Materials

The following supporting information can be downloaded at https://www.mdpi.com/article/10.3390/nu18111732/s1. Figure S1: Conceptual framework for the secondary analysis of DII/E-DII and cardiovascular outcomes; Table S1: Model-estimated marginal means of cardiovascular outcomes by E-DII tertile; Table S2: Within-person changes in DII and E-DII across study periods; Table S3: Between-sequence comparison of DII and E-DII change across intervention periods; Table S4: Sensitivity analysis excluding waist circumference from adjusted models: associations between DII tertiles and cardiovascular outcomes.
