# Supplementary material for: Dietary Inflammatory Index and Cardiovascular Disease Risk in Australian Adults: A Secondary Analysis of the OLIVAUS Trial
Source: Nutrients. 2026 May 28;18(11):1732. doi: 10.3390/nu18111732 (PMC13258959; doi:10.3390/nu18111732)
Supplement: Supplementary file 1 [file nutrients-18-01732-s001.zip › nutrients-4309137-supplementary.pdf]

Supplementary Material

Table S1. Model-estimated marginal means of cardiovascular outcomes by E-DII tertile

Cardiovascular outcomes	Tertile 1 (Low E-DII E-DII≤-1.32)	Tertile 2 (Medium E-DII -1.32<E-DII≤-0.414)	Tertile 3 (High E-DII E-DII>0.414)
Peripheral SBP (mmHg)	120 (117-123)	120 (116-123)	122 (119-126)
Peripheral DBP (mmHg)	69.0 (66.7-71.2)	68.8 (66.7-70.9)	70.9 (68.2-73.6)
Central SBP (mmHg)	107 (104-109)	106 (103-109)	109 (106-113)
Central DBP (mmHg)	69.7 (67.4-71.9)	69.5(67.4-71.7)	71.3 (68.6-74.0)
Triglycerides (mmol/L)	0.89 (0.72-1.07)	0.92 (0.76-1.09)	1.05 (0.84-1.26)
Total cholesterol (mmol/L)	4.97 (4.69-5.25)	4.98 (4.71-5.26)	5.10 (4.78-5.42)
HDL-cholesterol (mmol/L)	1.57 (1.47-1.67)	1.61 (1.51-1.70)	1.59 (1.47-1.70)
LDL-cholesterol (mmol/L)	3.02 (2.78-3.26)	2.98 (2.75-3.22)	3.08 (2.81-3.34)
Oxidised LDL (mU/mL)	71.7 (65.1-78.2)	73.3 (67.4-79.2)	72.4 (63.6-81.2)
HDL-Cholesterol efflux (%)	52.5 (51.1–53.9)	53.5 (52.1–54.8)	52.6 (51.0–54.2)
hsCRP (mg/L)	1.14 (0.55-1.72)	1.25 (0.71-1.78)	0.77 (0.01-1.53)

[1] Abbreviations: SBP, systolic blood pressure; DBP, diastolic blood pressure; HDL, high density lipoprotein; LDL, low density lipoprotein; E-DII, Energy-Adjusted dietary inflammatory index; hsCRP, high-sensitivity C-reactive protein; [2] Values are estimated marginal means (95% confidence intervals) from linear mixed-effects models adjusted for intervention, period, sequence, age, sex, and waist circumference, averaged across the study period.

Overall, peripheral and central blood pressure measures were broadly similar across tertiles, although participants in the highest E-DII tertile exhibited modestly higher peripheral and central systolic and diastolic blood pressure compared with those in the lowest tertile. Lipid profiles showed a graded trend across increasing E-DII, with higher triglyceride and total cholesterol concentrations observed in the highest versus lowest tertile. LDL-cholesterol and oxidised LDL concentrations were comparable across tertiles, while HDL-cholesterol levels showed minimal variation. HDL-cholesterol efflux capacity was also similar across E-DII tertiles, with no clear dose–response pattern evident. hsCRP concentrations were also broadly similar across E-DII tertiles, with no clear dose–response pattern, although values were slightly lower in the highest tertile than in the lower tertiles.

Table S2. Within-person changes in DII and E-DII across study periods

Measure	Period 1			Period 2		
	T1 (Mean ± SD)	T3 (Mean ± SD)	P	T4 (Mean ± SD)	T6 (Mean ± SD)	p
DII	−0.810 ± 1.55	−0.976 ± 1.46	0.484	−0.500 ± 1.80	−0.642 ± 1.32	0.576
E-DII	−1.174 ± 1.13	−0.288 ± 1.36	< 0.001*	−0.782 ± 1.66	−0.186 ± 1.32	0.021*

[1] Abbreviations: DII, dietary inflammatory index; E-DII, Energy-Adjusted dietary inflammatory index; [2] *Indicates statistically significant inter-tertile differences, $p < 0.05$.

Table S3. Between-sequence comparison of DII and E-DII change across intervention periods

Changes		HPOO-LPOO (Mean ± SD)	LPOO-HPOO (Mean ± SD)	p
DII change	Period 1	−0.0629 ± 1.577	−0.282 ± 1.68	0.646
	Period 2	−0.0534 ± 0.998	−0.230 ± 2.11	0.730
E-DII change	Period 1	0.9958 ± 1.628	0.761 ± 1.35	0.597
	Period 2	0.4355 ± 1.211	0.756 ± 1.94	0.524
DII total change		0.0200 ± 0.906	−0.264 ± 1.40	0.438
E-DII total change		0.7395 ± 1.060	21, 0.768 ± 1.29	0.938

[1] Abbreviations: HPOO, high polyphenol olive oil; LPOO, low polyphenol olive oil; DII, dietary inflammatory index; E-DII, Energy-Adjusted dietary inflammatory index; [2] *Indicates statistically significant inter-tertile differences, $p < 0.05$.
