# Supplementary material for: Dietary Inflammatory Index and Cardiovascular Disease Risk in Australian Adults: A Secondary Analysis of the OLIVAUS Trial
Source: Nutrients. 2026 May 28;18(11):1732. doi: 10.3390/nu18111732 (PMC13258959; doi:10.3390/nu18111732)
Supplement: Supplementary file 1 [file nutrients-18-01732-s001.zip › Supplementary Table S4.pdf]

Supplementary Table S4. Sensitivity analysis excluding waist circumference from adjusted models: associations between DII tertiles and cardiovascular outcomes.

Cardiovascular outcomes	Medium DII vs Low DII β (95% CI)	p-value	High DII vs Low DII β (95% CI)	p-value
Peripheral SBP (mmHg)	0.31 (−2.30, 2.92)	0.813	1.26 (−1.63, 4.14)	0.391
Peripheral DBP (mmHg)	0.03 (−1.94, 1.99)	0.979	1.29 (−0.87, 3.45)	0.239
Central SBP (mmHg)	0.71 (−1.68, 3.11)	0.558	1.26 (−1.37, 3.90)	0.345
Central DBP (mmHg)	0.17 (−1.78, 2.12)	0.864	1.20 (−0.95, 3.34)	0.272
Triglycerides (mmol/L)	0.01 (−0.15, 0.16)	0.899	0.00 (−0.17, 0.17)	0.963
Total cholesterol (mmol/L)	0.08 (−0.10, 0.27)	0.366	−0.04 (−0.24, 0.16)	0.695
HDL-cholesterol (mmol/L)	0.05 (−0.02, 0.11)	0.159	0.04 (−0.04, 0.11)	0.317
LDL-cholesterol (mmol/L)	0.07 (−0.07, 0.21)	0.345	−0.05 (−0.21, 0.10)	0.497
Oxidised LDL (mU/mL)	−3.72 (−11.41, 3.96)	0.340	3.27 (−4.82, 11.37)	0.426
HDL-cholesterol efflux (%)	−0.10 (−1.06, 0.87)	0.844	−0.16 (−1.24, 0.91)	0.764
hsCRP (mg/L)	0.14 (−0.50, 0.78)	0.671	0.49 (−0.20, 1.18)	0.160

Abbreviations: SBP, systolic blood pressure; DBP, diastolic blood pressure; HDL, high density lipoprotein; LDL, low density lipoprotein; DII, dietary inflammatory index; hsCRP, high-sensitivity C-reactive protein; β = adjusted mean difference for medium vs low DII and high vs low DII; Models adjusted for age and gender
