# Supplementary figures and images for: Dietary Inflammatory Index and Cardiovascular Disease Risk in Australian Adults: A Secondary Analysis of the OLIVAUS Trial
Source: Nutrients. 2026 May 28;18(11):1732. doi: 10.3390/nu18111732 (PMC13258959; doi:10.3390/nu18111732)

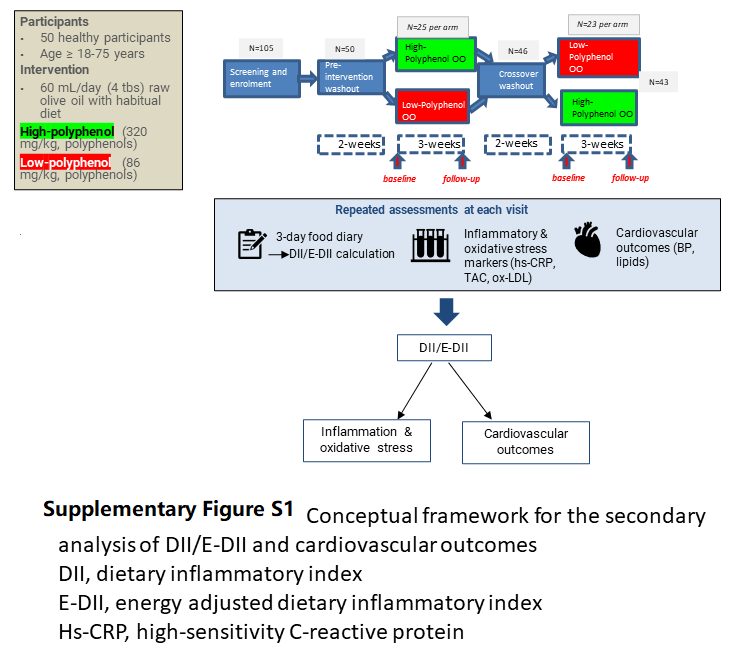

Supplement: Supplementary file 1 [file nutrients-18-01732-s001.zip › Supplementary Figure S1.PNG]
